# Effects of dry needling on recovery, neuromuscular function, and injury outcomes in sports athletes: a systematic review targeting the neck and related regions

**DOI:** 10.3389/fpubh.2026.1813780

**Published:** 2026-04-13

**Authors:** Gracjan Olaniszyn, Irenusz Ryszkiel, Robert Trybulski, Cyprian Olchowy, Karol Pilis

**Affiliations:** 1 Faculty of Medicine, Katowice Business University, Katowice, Poland; 2 Olaniszyn Physiotherapy Centre, Racibórz, Poland; 3 College of Medical Sciences, Medical University of Silesia, Katowice, Poland; 4 Provita Medical Centre, Żory, Poland; 5 Department of Cardiovascular Diseases, Collegium Medicum, Jan Dlugosz University, Czestochowa, Poland; 6 Department of Health Sciences and Physiotherapy, Collegium Medicum, Jan Dlugosz University, Czestochowa, Poland

**Keywords:** dry needling, needling, sports injuries, sports medicine, sports recovery

## Abstract

**Background:**

Dry needling (DN) is used in sports medicine for myofascial pain, injury and recovery, but athlete-specific effects over time are uncertain. Objective: To synthesize evidence on DN applied to cervical and related upper-quarter regions in athletes, distinguishing (i) symptomatic athlete trials from (ii) post-exertion recovery trials, and to summarize injury-related outcomes only when prospectively reported.

**Data sources:**

PubMed, Scopus, and Web of Science searched 11 Feb 2026. Study selection: Trials/prospective studies in athletes comparing DN (alone or add-on) with sham/no intervention or active comparators.

**Methods:**

Two authors independently screened/extracted data and assessed risk of bias (RoB 2; ROBINS-I).

**Results:**

Eight studies were included. In symptomatic overhead/throwing athletes, DN improved pain and shoulder ROM immediately versus no treatment in one trial, while several trials showed no differences versus active comparators or sham. Recovery trials showed inconsistent differences in hemodynamic indices and inflammatory markers, and their short follow-up and proxy endpoints limit inference about next-session performance, training continuity, or injury risk.

**Conclusion:**

DN may provide short-term symptom modulation in sport athletes. Evidence for consistent recovery/readiness enhancement, performance benefit, or injury prevention remains highly uncertain and is constrained by proxy outcome selection and short time windows. Registration: OSF osf.io/fsg6w (11 February 2026).

**Systematic review registration:**

OSF (Open Science Framework; ID: osf.io/fsg6w (11 February 2026).

## 1 Introduction

Dry needling (DN) is a minimally invasive intervention in which a thin, solid filiform needle is inserted through the skin to mechanically and neurophysiologically stimulate myofascial trigger points and related neuromusculoskeletal tissues, with the clinical intent of reducing symptoms and improving movement (1). DN is defined as intramuscular filiform needle insertion intended to mechanically stimulate a clinically targeted myofascial trigger point (or closely related myofascial tissue) and to evoke needle-driven sensory input, typically delivered via either repeated pistoning/“fast-in fast-out” insertions or brief needle retention/*in situ* techniques (with or without an elicited local twitch response) (2). However, the construct of discrete trigger points as primary causal pain generators and the specificity of trigger-point targeted mechanisms are debated across pain science and sports medicine, thus, observed effects may reflect a mixture of local tissue perturbation, non-specific neuromodulation, and contextual (expectation/meaning) effects rather than a single trigger-point pathway (3). Within the trigger-point framework, DN is commonly conceptualized as an approach to decrease ongoing peripheral nociceptive input from myofascial trigger points and, through peripheral–central interactions, facilitate improvements in pain, range of motion, and muscle activation patterns (4). Because DN intentionally perturbs sensitive myofascial tissue, transient post-needling soreness is a frequent short-term effect (typically resolving within 72 h) and is clinically relevant when DN is applied close to training and competition schedules (5).

Neck pain is prevalent in athletic populations, with systematic evidence indicating substantial prevalence estimates across sports and time horizons, supporting the clinical importance of cervical-region symptom management in athletes (6). This burden is clinically relevant to the overhead athletes and paraathletes (e.g., handball; volleyball; combat sports), where upper-quarter loading demands and sport-specific exposures are common and neck/upper-quarter pain complaints have been documented (6, 7). In overhead athletes, upper trapezius myofascial trigger points have been associated with altered scapular kinematics and muscle activation patterns during arm elevation after a fatigue task, linking cervical/periscapular myofascial dysfunction to fatigue-sensitive neuromuscular control (8). However, association does not establish that trigger points are causal factors of altered kinematics or that needling will normalize movement patterns. In parallel, delayed-onset muscle soreness (DOMS) is a common consequence of unaccustomed or high-load eccentric exercise and can impair perceived readiness and task execution in the short term, making recovery-oriented interventions a persistent priority in sports medicine and performance contexts (9). At the same time, DOMS and its inflammatory/repair processes also reflect normal adaptation to eccentric loading, therefore, DOMS reduction is not inherently synonymous with better adaptation and is most relevant when soreness meaningfully degrades skill quality, increases perceived effort, or constrains planned training exposure (9, 10).

Contemporary synthesis specific to neck pain indicates low-to-moderate certainty evidence that DN targeting trigger points can improve pain intensity and pain-related disability in the short term, while mid-term effects and several mechanistic/functional outcomes remain less consistent (11). Randomized evidence in the upper trapezius region further suggests that DN and comparator trigger-point techniques can both yield clinically meaningful improvements over follow-up, reinforcing that DN is a credible option within broader myofascial management strategies for cervical and pericervical disorders (12). In sport-focused synthesis, DN appears more consistently favorable for pain and perceived muscle stiffness than for direct athletic performance endpoints, indicating a potential dissociation between symptom modulation and performance transfer (13).

Empirical sport-adjacent trials illustrate heterogeneity in outcomes, with DN showing immediate flexibility-related effects in some contexts yet failing to reduce post-event soreness/cramping in others, underscoring the need to clarify indications, timing, and target tissues for recovery applications (14, 15).

Despite expanding DN literature, fatigue and recovery in sport are multidimensional constructs with limited mechanistic clarity linking workload-derived fatigue to injury risk, creating challenges for interpreting DN effects beyond short-term symptom change (16). Broader recovery meta-analytic evidence across modalities emphasizes measurable impacts on DOMS and perceived fatigue, yet DN is not consistently represented or evaluated with standardized recovery and readiness measures in these frameworks (17). Sport-specific DN synthesis also highlights underrepresentation of elite/world-class cohorts and a lack of long-term, sport-relevant endpoints (e.g., next-session performance, validated readiness-to-train measures, training/competition availability, time-loss injury, recurrence, and return-to-play time), which limits translation to high-performance decision-making (13). Additionally, the high frequency of short-lived post-needling soreness can be a practical barrier to deploying DN as a recovery intervention, making the balance of benefit versus short-term adverse effects particularly salient in competitive calendars (5).

Based on these reasons, this systematic review aimed to evaluate the effectiveness of DN applied to cervical and adjacent peripheral regions (e.g., upper trapezius/shoulder girdle and relevant limb muscles) in two distinct athlete-relevant contexts: (1) cervical/upper-quarter symptom management in athletes with regional pain/overuse presentations, and (2) post-exertion recovery/readiness applications where DN was applied to upper-quarter muscles engaged in sport-specific loading. We did not intend to evaluate whole-body global recovery interventions unrelated to cervical/upper-quarter targets. For clarity, recovery is defined as the trajectory back toward pre-exercise capacity and tolerable symptoms after a defined exertional bout, whereas readiness reflects the athlete’s ability to tolerate the next planned session with acceptable performance and symptoms. Accordingly, proxy outcomes are interpreted hierarchically considering validated readiness scales and next-session performance/availability are closest to the applied construct, whereas isolated physiological markers (e.g., hemodynamics, cytokines, muscle thickness) are treated as correlates without established directionality for better recovery. Injury outcomes (e.g., incidence, recurrence, time-loss, return-to-play time) were extracted when reported, but we did not assume that intermediate outcomes (e.g., pain, range of motion, ROM, or pressure pain threshold, PPT) validate injury-risk modification in the absence of prospective injury surveillance.

We aimed to synthesize evidence on DN effects on injury-related outcomes (incidence, recurrence, time-loss, symptom exacerbation) and on intermediate risk-relevant measures (pain, stiffness, range of motion, neuromuscular function, and performance proxies) when reported in athletic samples. Finally, we aimed to characterize intervention parameters (timing relative to training/competition, dosage, target tissues, elicitation of local twitch responses, comparator types) and document adverse events to inform clinically implementable upper-quarter recovery and injury-risk management strategies in sport.

## 2 Methods

This protocol was developed *a priori* and was reported in line with the PRISMA 2020 statement and expanded checklist recommendations (18). The study protocol was preregistered in the Open Science Framework (OSF; ID: osf.io/fsg6w; registered on 11 February 2026).

### 2.1 Eligibility criteria

Studies were selected using a PICO framework. The population included competitive or recreational athletes of any age, sex, and sport, including healthy athletes and those with pain, fatigue-related symptoms, or sport-related musculoskeletal conditions. Studies with mixed samples were eligible only when athlete-specific data could be extracted separately.

The intervention included studies in which dry needling (DN; filiform needle insertion) was applied to the neck region and/or upper-quarter regions with a predefined theoretical link to cervical/neck outcomes, namely muscles within the cervical–scapulothoracic–glenohumeral kinetic chain that plausibly influence neck loading or upper-quarter movement tolerance (regional interdependence/biomechanical chain rationale), or upper-limb muscles explicitly targeted in post-exertion recovery studies when the exertional task predominantly involved the upper body and outcomes were framed as recovery/readiness proxies. Regions were considered in scope only when the study explicitly linked the target tissue to neck/upper-quarter symptoms, upper-quarter function, or upper-body recovery after exertion; otherwise, the study was excluded as anatomically out of scope.

DN could be delivered as a standalone intervention or within a multimodal program, provided the DN component was clearly described. Eligible comparators included sham/placebo needling, no intervention, usual care, wait-list control, or active comparators (e.g., manual therapy, stretching, exercise, soft-tissue techniques, taping, modalities, and injection-free trigger point techniques). Head-to-head comparisons of DN parameters (e.g., with vs. without local twitch response, different dosages) were also eligible.

Eligible outcomes included fatigue recovery/readiness outcomes (e.g., perceived fatigue, delayed-onset muscle soreness, recovery scales, training-readiness indices), prospectively measured injury-related outcomes when available (e.g., incidence, recurrence, time-loss, symptom flare/exacerbation, return-to-play time), and intermediate/mechanistic outcomes that may plausibly mediate symptom tolerance or short-term function (e.g., pain, stiffness, range of motion, neuromuscular performance, muscle function, proprioception, strength, power, sprint/throwing measures, and sport-specific performance proxies). Intermediate outcomes were not treated as evidence of injury-risk modification unless coupled with prospective injury surveillance. Studies were eligible even if injury outcomes were not reported, provided at least one symptom, function, or recovery/readiness outcome was reported.

Studies were eligible even if injury outcomes were not reported, provided at least one recovery or intermediate outcome was reported. Eligible study designs included randomized controlled trials (individual or cluster), quasi-randomized trials, non-randomized controlled trials, and prospective cohort studies. Case series/reports, cross-sectional studies, narrative reviews, editorials, and animal studies were excluded.

Full-text, peer-reviewed articles were eligible; conference abstracts were excluded unless a corresponding full report was obtainable. No date restrictions were applied. Studies in languages not readable by the team were translated for eligibility assessment and data extraction when feasible. Feasibility was defined *a priori* as access to a fluent speaker within the author team/network or a professional translation resource sufficient for screening and extraction.

### 2.2 Information sources

The following databases were searched on 11 February 2026: PubMed (MEDLINE interface); Web of Science Core Collection (Clarivate); and Scopus (Elsevier). In addition, reference lists of all included studies and of relevant prior systematic reviews identified during screening were checked (backward citation searching).

### 2.3 Search strategy

Search strategies were constructed around two core concepts: dry needling, and athletes/sport. Database-specific controlled vocabulary and syntax were adapted for each platform. No methodological search filters were applied. Anatomical keywords (e.g., “neck,” “cervical,” “upper trapezius”) were not included to maximize sensitivity because athlete DN studies may not consistently index the targeted body region in titles/abstracts/keywords and some recovery-oriented DN studies target upper-quarter muscles (e.g., pectoralis/deltoid/triceps) without explicitly labeling “neck/cervical” despite being within the pre-specified related regions scope. The following search strategy was implemented for titles, abstracts and keywords, or topic in the case of Web of Science:


$$\begin{array}{l} “dry\;{\text{needl}}^{*”}\left[\text{Title}/\text{Abstract}\right]\text{AND }{(}^{“}{\text{sport}}^{*”}\left[\text{Title}/\text{Abstract}\right]\;\\ {\text{OR}}^{“}\text{athletic }{\text{performance}}^{*”}\left[\text{Title}/\text{Abstract}\right]\\ {\text{OR}}^{“}{\text{exercise}}^{*”}\left[\text{Title}/\text{Abstract}\right]\;{\text{OR}}^{“}\text{athletic }{\text{injur}}^{*”}\left[\text{Title}/\text{Abstract}\right]). \end{array}$$

### 2.4 Selection process

All retrieved records were exported from each database and de-duplicated using reference management software (Endnote online) and manual verification. Manual verification was performed independently by two authors (GO, RT). Disagreements were resolved by consensus and recorded in a de-duplication decision log. If consensus was not achieved, a third author (KP) adjudicated. Full texts were obtained for all records judged potentially eligible by either author.

### 2.5 Data collection process

Two authors (GO and RT) independently extracted data using a standardized extraction form. Disagreements were resolved by consensus and, when required, third-author arbitration. No missing data were observed. When values were only available in graphs and numerical extraction was required, we estimated them from the figures using WebPlotDigitizer.

### 2.6 Data items

Data were sought for all outcomes within each eligible outcome domain, across all reported measurement instruments and time points. Outcomes were grouped as: (i) Primary outcome domains, namely, fatigue recovery/readiness (e.g., perceived fatigue scales, recovery indices, delayed onset muscle soreness (DOMS), perceived muscle stiffness, readiness-to-train/compete, and related psychophysiological recovery markers where available) and injury-related outcomes (incidence, recurrence, time-loss, symptom exacerbation, return-to-play time, or medically attended injury events); and (ii) secondary/intermediate outcome domains, namely pain intensity and disability (where relevant to recovery), range of motion (ROM), and neuromuscular performance (e.g., strength, power, rate of force development, endurance). When multiple measures/time points were reported for the same domain, the following decision rule was applied: (i) prioritize the most sport-relevant and validated measure; (ii) prioritize time points aligned with short-term recovery windows (≤72 h) for recovery outcomes; (iii) additionally extract the longest follow-up for durability of effects; and (iv) extract all time points when feasible and report them in structured tables. Sport-relevant was classified as conceptual proximity to athlete decision-making (e.g., next-session performance, readiness status, availability).

Additionally, the following variables were extracted: study design; setting and sport; sample characteristics (age, sex, training level, injury status); DN parameters (target muscles/regions, number of sessions, needling technique, elicitation of local twitch response when stated, session duration, clinician qualifications); comparator details; co-interventions; timing relative to training/competition; and adverse events.

### 2.7 Study risk of bias assessment

Risk of bias was assessed independently by two authors (GO and RT). For randomized trials, the Cochrane RoB 2 tool was used (domains: randomization process, deviations from intended interventions, missing outcome data, measurement of the outcome, selection of the reported result). For non-randomized studies, ROBINS-I was used (domains: confounding, selection, classification of interventions, deviations, missing data, measurement, selection of reported result). Disagreements were resolved by consensus/third author. Overall risk-of-bias judgments were assigned according to the tool guidance and were presented by domain and overall rating for each study.

### 2.8 Effect measures and synthesis methods

For continuous outcomes, mean difference (MD) was preferred when the same instrument/scale was used, otherwise standardized mean difference (SMD; Hedges g) was extracted when different instruments assessed the same construct. For time-to-event outcomes, hazard ratios (HR) were extracted when available. For crossover and other within-subject (paired) designs, we preferentially extracted the authors’ paired effect estimates (within-subject MD/SMD) and corresponding SE/CI/*p*-values.

Studies were grouped for synthesis by: (i) population type (healthy vs. symptomatic/injured athletes), (ii) anatomical target (neck/cervical vs. pericervical/upper quarter vs. other peripheral muscles explicitly linked to the objectives), (iii) timing relative to exertion (pre-, immediate post-, 24–72 h post-, or longer), and (iv) comparator type (sham/no intervention vs. active comparator vs. add-on designs). For multi-arm trials, we avoided double-counting shared comparator groups by selecting the most clinically relevant contrast per synthesis question (e.g., DN vs. sham/no intervention for absolute effects; DN vs. active standard for incremental effects). Where multiple DN arms were present (e.g., different DN doses), we reported each arm descriptively but did not aggregate them against a single shared comparator in the same narrative contrast. For add-on designs (DN and co-intervention vs. co-intervention alone), effects were interpreted explicitly as incremental (added-value) effects of DN rather than absolute effects of DN in isolation.

## 3 Results

### 3.1 Study selection

**Figure 1:**
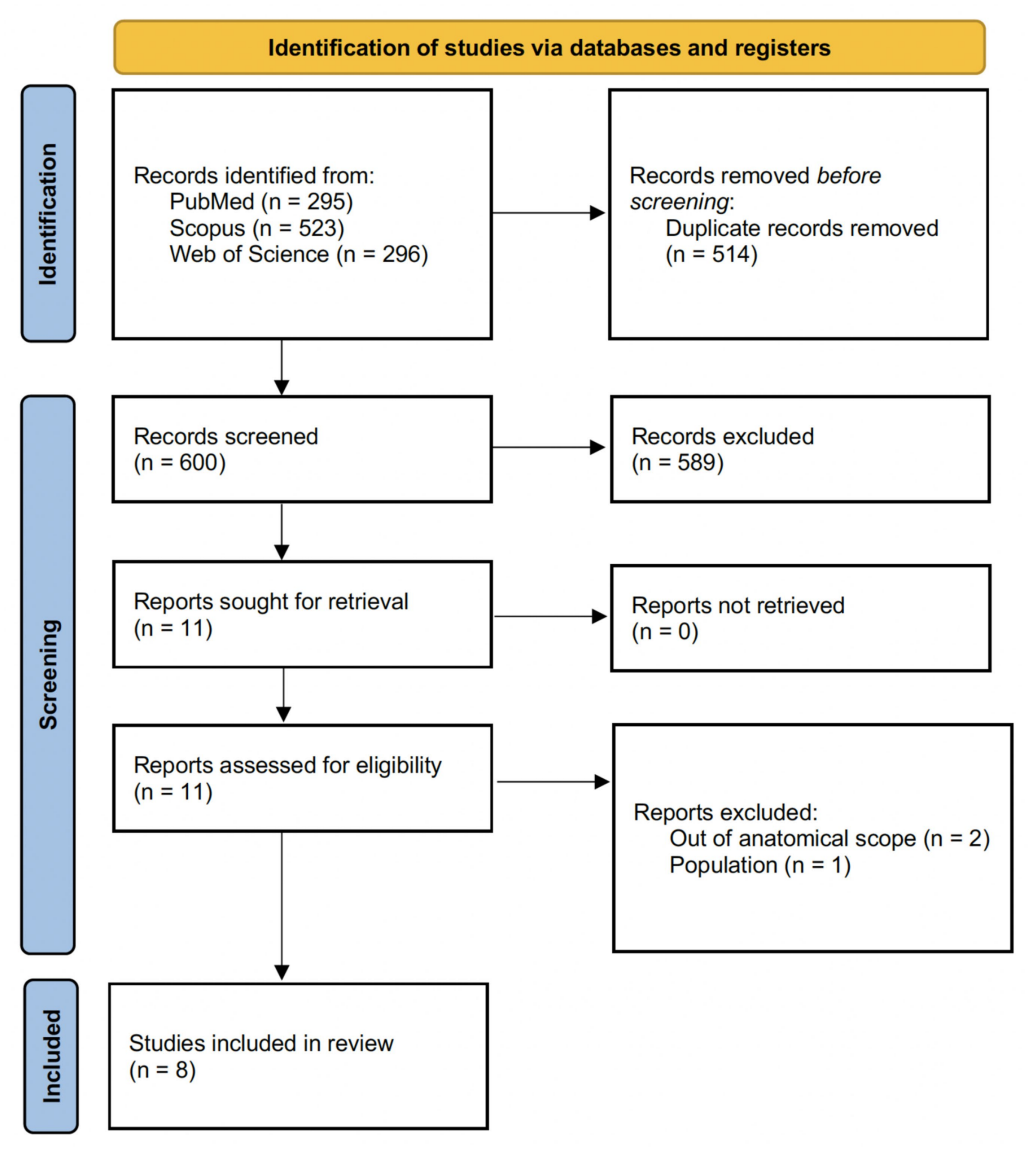
PRISMA flow diagram.

During database searching, we identified 1,114 records (PubMed *n* = 295; Scopus *n* = 523; Web of Science *n* = 296). After removing 514 duplicates, 600 records were screened by title/abstract, of which 589 were excluded. We sought to retrieve 11 full-text reports, and all were successfully retrieved. Following full-text eligibility assessment (*n* = 11), three reports were excluded [out of anatomical scope, *n* = 2 (19, 20); population not meeting eligibility criteria, *n* = 1 (21)]. Overall, eight studies met the inclusion criteria and were included in the systematic review. The study identification, screening, eligibility assessment, and inclusion process is summarized in the flow diagram (Figure 1).

### 3.2 Characteristics of the included studies

Table 1 summarizes study characteristics. Most studies used an RCT design (*n* = 5), with crossover recovery trials (*n* = 2) and one single-group pre–post study (*n* = 1). The evidence base was predominantly focused on injury/overuse or pain interventions (*n* = 6) rather than recovery/fatigue applications (*n* = 2). Athlete samples were most frequently from handball (*n* = 3), followed by mixed overhead-sport athletes (*n* = 2), Paralympic powerlifting (*n* = 2), and combat sports (*n* = 1), thus sport may be interpreted as a contextual modifier of both baseline symptoms and the practical value/risks of DN timing, rather than as a neutral descriptor. Comparator selection was mainly active comparators (*n* = 5), with fewer studies using sham DN (*n* = 1), no-intervention control (*n* = 1), or no comparator (*n* = 1). Outcome assessment was largely short-term (immediate and/or ≤1 week), while recovery trials monitored multiple post-exercise/recovery timepoints up to 60 min (*n* = 1) or 48 h (*n* = 1). Accordingly, these time windows are insufficient to infer durable functional change, training continuity, or injury-risk modification. In the crossover recovery trials (Paralympic powerlifting), conditions were delivered across separate weeks. Aidar et al. (22) reported weekly visits with a 7-day washout, whereas Santos et al. (23) implemented the three recovery conditions across weeks 2–4.

**Table 1:** Study designs and methodological characteristics.

Study	Context	Injury type	Design	N (arms)	Age	Sex	Sport / level	Intervention vs. comparator	Timepoints	Tests	Outcomes
Kużdzał et al. (35)	Sports injury/pain intervention	Acute unilateral myofascial neck pain (upper trapezius MTrP)	Single-blind parallel RCT (repeated measures)	30 (MTDN *n* = 15; CG *n* = 15)	MTDN 24.3 ± 3.2; CG 26.6 ± 4.4 (range 18–35)	23 M/7F (MTDN 11 M/4F; CG 12 M/3F)	Combat sports (MMA, Judo, BJJ); Tier 2 (national)	MTDN (DN + manual therapy) vs. CG (sham needle + manual therapy)	T0 baseline; T1 + 5 min post S1; T2 + 5 min post S3; T3 72h post S3	MyotonPRO; algometer; handheld dynamometer; electronic goniometer	MT, MS, PPT, Fmax, ROM (lat flex, rotation)
Kheradmandi et al. (29)	Sports injury/overuse condition intervention	Shoulder pain in overhead athletes with scapular dyskinesia	Single-blind RCT	40 (DN + MT *n* = 20; MT *n* = 20)	DN 32.2 ± 8.35; Control 31.8 ± 6.85	15 M/25F (overall)	Overhead athletes (mixed sports; ≥1y overhead activity)	DN + manual therapy vs. manual therapy alone	T0 baseline; T1 1 day after last session	NRS; PPT (algometer); DASH; SDT; LSST (3 positions)	Pain, PPT, disability, dyskinesia
Ceballos-Laita et al. (24)	Sports injury/overuse condition intervention	Shoulder pain during throwing; GIRD ≥15°; active teres major MTrP	Randomized single-blind controlled trial	30 (DN *n* = 15; control *n* = 15)	Overall 22.39 ± 3.73; DN 22.47 ± 3.04; control 22.31 ± 4.37	30 M/0F	Elite/professional handball	US-guided DN (teres major) vs. no intervention (rest same duration)	T0 baseline; T1 immediate post	NPRS; inclinometer ROM; handheld dynamometer strength; inclinometer extensibility	Pain, IR/ER ROM, strength (IR/ER), extensibility, GIRD/ERG
Kamali et al. (28)	Sports injury/overuse condition intervention	Unilateral shoulder impingement syndrome (Neer/Hawkins +); active UT + ISP MTrPs	Single-blind RCT (active-comparator)	40 (UT DN *n* = 21; ISP DN *n* = 19)	36 ± 16 (range 18–60)	20 M/20F	Overhead athletes (semi-elite; throwers/swimmers/volleyball/basketball)	UT DN (direct) vs. ISP DN (remote)	T0 baseline; T1 3 days after last session	VAS pain; PPT (algometer); DASH	Pain, PPT, disability
Aidar et al. (22)	Recovery process - Post-strength training hemodynamic recovery (bench press ST)		Randomized crossover (3 recovery conditions)	12 (within-subject: PR vs. CW vs. DN)	25.40 ± 3.30	12 M/0F	Paralympic powerlifting; national level (top 10)	DN vs. PR vs. CW (15-min recovery each)	Pre-ST; post-ST; post-recovery 0–60 min (multiple moments)	Automated BP monitor; HR; derived DP and MVO2	SBP, DBP, MBP, HR, DP, MVO2
Santos et al. (23)	Recovery process - Exercise-induced muscle damage (eccentric overload bench press)		Crossover (3 recovery methods over 3 weeks)	12 (within-subject: PR vs. DN vs. CWI)	25.4 ± 3.3	12 M/0F	Paralympic powerlifting; national level (top 10)	DN vs. PR vs. CWI	Pre; +15 min (post recovery); +2 h; +24 h; +48 h	PPT algometer; ultrasound muscle thickness; cytokines (IL-2, IL-4, IFN-γ); MIF force sensor	PPT, muscle thickness, cytokines, MIF
Ceballos-Laita et al. (32)	Sports injury/overuse condition intervention	Shoulder pain during throwing; GIRD >15°; active teres major MTrP	RCT with blinded examiners	30 (DN *n* = 15; DF *n* = 15)	DN 25.47 ± 4.99; DF 26.20 ± 5.91	30 M/0F	Elite handball	DN (teres major) vs. DF (teres major)	T0 baseline; T1 post; T2 1-week follow-up	MyotonPRO; VAS; digital inclinometer (IR/ER/HA ROM, extensibility)	Stiffness, tone, pain, ROM (IR/ER/tROM/HA), extensibility
Pavlović et al. (25)	Sports pain condition intervention	Myofascial pain syndrome (multi-region MTrPs)	Repeated-measures (pre–post)	50 (single group)	M 23.0 ± 2.4; F 22.7 ± 1.9 (overall NR)	38 M/12F	Mixed sports; ≥5y competitive (football, basketball, volleyball, handball, combat, swimming, tennis, track&field)	Dry needling only (no comparator)	T0 pre first session; T1 immediate post last session (4th)	SF-36	Quality of life (SF-36 dimensions)

MTrP: myofascial trigger point; RCT: randomized controlled trial; MTDN: manual therapy + dry needling; CG: control group; BJJ: Brazilian jiu-jitsu; MMA: mixed martial arts; DN: dry needling; S1: session 1; S3: session 3; Fmax: maximal isometric force; PPT: pressure pain threshold; ROM: range of motion; MS: muscle stiffness; MT: muscle tone; DASH: Disabilities of the Arm, Shoulder and Hand questionnaire; NRS: Numeric Rating Scale; SDT: Scapular Dyskinesis Test; GIRD: glenohumeral internal rotation deficit; US: ultrasound; NPRS: Numeric Pain Rating Score; ERG: external rotation gain; IR: internal rotation; ISP: infraspinatus; UT: upper trapezius; VAS: visual analog scale; ST: strength training; CW: cold water; PR: passive recovery; MVO2: myocardial oxygen consumption (estimated); BP: blood pressure; DP: double product; HR: heart rate; DBP: diastolic blood pressure; MBP: mean blood pressure; SBP: systolic blood pressure; CWI: cold-water immersion; IFN-γ: interferon gamma; MIF: maximal isometric force (bench press); IL: interleukin; DF: diacutaneous fibrolysis; HA: horizontal adduction; tROM: total range of motion; NR: not reported; SF-36: 36-item Short Form Health Survey.

Table 2 details DN application characteristics across the included studies. Across injury/overuse studies, the most commonly reported outcomes were pain intensity measures (VAS/NRS/NPRS) (*n* = 4) and PPT (*n* = 4), with ROM/mobility outcomes (*n* = 3) and DASH disability/function (*n* = 2) reported less frequently. Two studies quantified muscle mechanical/viscoelastic variables using Myoton-based measures (*n* = 2), and strength/force outcomes were included in *n* = 3. The recovery-focused studies primarily evaluated either hemodynamic recovery (BP/HR/DP/MVO₂) (*n* = 1) or exercise-induced muscle damage markers (e.g., cytokines and ultrasound muscle thickness) (*n* = 1).

**Table 2:** Dry needling applications.

Study	Frequency/total	Target region	Muscles	Technique	Needle	Dose/session	Comparator	Provider/adverse events
Kużdzał et al. (35)	3 sessions; q3d (3-day interval)	Upper trapezius (neck/upper quarter)	Upper trapezius active MTrP	DN to painful active MTrP; LTR sought (≤5 insertions); ~1 min	SOMA sterile 0.30 × 0.30 mm	Single needle per MTrP; then IC ≤ 90s + 3 friction rubs (3–5 min)	Telescopic sham (no skin penetration) + same manual therapy	Physiotherapist; adverse reactions recorded (counts NR)
Kheradmandi et al. (29)	3 sessions; q3d	Shoulder/upper quarter	Subscapularis, pec minor, serratus ant., upper/lower trap (if MTrPs)	DN after scapular mobilization (LTR/needle details NR)	NR	NR	Same scapular mobilization only (no DN)	Expert PT; assessor blinded; AE NR
Ceballos-Laita et al. (24)	1 session (single treatment)	Posterior shoulder/upper quarter	Teres major active MTrP	US-guided DN; Hong fast-in fast-out; LTR visualized; until LTR extinct	0.30 × 50 mm stainless, single-use	Needling until LTR extinction (time NR)	No intervention (prone rest same duration)	Experienced PT (>7y DN); sonographer (>10y); AE NR
Kamali et al. (28)	3 sessions; q2d (2-day interval)	Upper quarter	UT group: upper trapezius MTrP; ISP group: infraspinatus MTrP	Pistoning to MTrP until no more LTRs; points marked	Acupuncture needle 0.2 × 50 mm (guide tube)	Needling until LTRs ceased (time/insertions NR)	Active comparator (UT DN vs. ISP DN)	Trained PT; AE reporting NR
Aidar et al. (22)	3 conditions; 1 session/condition; weekly visits (7-day washout)	Upper body/upper arm	Pectoralis major, anterior deltoid, triceps brachii	In situ DN; perpendicular insertion; held 5 min; no manipulation	Stainless sterile monofilament 0.25 × 40 mm	Needles placed at each target muscle; total DN recovery period 15 min	Passive seated 15 min; cold-water immersion 15 °C up to neck for 15 min	Trained physiotherapist; AE NR
Santos et al. (23)	3 conditions; 1 session/condition over weeks 2–4 (crossover)	Upper body/upper arm	Pectoralis major, anterior deltoid, triceps brachii	In situ DN; perpendicular insertion; held 5 min; no manipulation	Stainless sterile monofilament 0.25 × 40 mm	Needles placed at each target muscle; recovery period 15 min	Passive seated 15 min; CWI 11–15 °C up to neck for 15 min	Trained physiotherapist; AE NR
Ceballos-Laita et al. (32)	1 session (single treatment)	Posterior shoulder/upper quarter	Teres major active MTrP	Fast-in fast-out DN with multiple insertions until no LTR; hemostasis compression after	0.25 × 50 mm acupuncture needle	Needling until LTRs ceased; ≤20 min total session time (both groups)	Diacutaneous fibrolysis (hook) around teres major (intermuscular septa)	Expert PT (>10y instrumental techniques); AE NR
Pavlović et al. (25)	4 sessions; 5–7 days apart	Multi-region (upper quarter + other regions)	55 muscles treated (e.g., trapezius, infraspinatus, deltoid, levator scapulae, pec major/minor, triceps/biceps, plus lower-limb/trunk)	Trigger point DN; perpendicular insertion; superficial or deep; needle retained 10–20 min with slight repositioning	Sterile acupuncture needle (size NR)	Needle retention 10–20 min; #needles/points NR	None	Licensed DN therapist (≥10y); side effects discussed (pain/bleeding/bruising); AE reporting NR

DN: dry needling; MTrP: myofascial trigger point; LTR: local twitch response; US: ultrasound; IC: ischemic compression; CG: control group; MTDN: manual therapy + dry needling; UT: upper trapezius; ISP: infraspinatus; q2d: every 2 days; q3d: every 3 days; PR: passive recovery; CW: cold water; CWI: cold-water immersion; BP: blood pressure; AE: adverse events; NR: not reported.

### 3.3 Risk of bias assessment

Table 3 summarizes the RoB 2 judgments for the seven randomized studies. Overall risk of bias was judged as “some concerns” in 6/7 studies (85.7%) and “high” in 1/7 studies (14.3%). By domain, Domain 1 (randomization process) was judged as low risk in 1/7 (14.3%) and some concerns in 6/7 (85.7%); Domain 2 (deviations from intended interventions) was judged as some concerns in 7/7 (100%); Domain 3 (missing outcome data) was judged as low risk in 7/7 (100%); Domain 4 (measurement of the outcome) was judged as low risk in 3/7 (42.9%), some concerns in 3/7 (42.9%), and high risk in 1/7 (14.3%); and Domain 5 (selection of the reported result) was judged as low risk in 1/7 (14.3%) and some concerns in 6/7 (85.7%). The only trial rated “high” overall RoB (24) is also the study reporting the largest immediate between-group differences versus no-intervention control (pain during throwing and ROM outcomes), whereas trials with some concerns more commonly reported null or small incremental differences versus active comparators or sham.

**Table 3:** Risk of bias assessment in randomized trials.

Study	Domain 1: Randomization	Domain 2: Deviations	Domain 3: Missing data	Domain 4: Measurement	Domain 5: Selective reporting	Overall RoB
Aidar et al. (22)	Some concerns	Some concerns	Low	Low	Some concerns	Some concerns
Ceballos-Laita et al. (24)	Some concerns	Some concerns	Low	High	Some concerns	High
Ceballos-Laita et al. (32)	Some concerns	Some concerns	Low	Low	Low	Some concerns
Kamali et al. (28)	Some concerns	Some concerns	Low	Some concerns	Some concerns	Some concerns
Kheradmandi et al. (29)	Some concerns	Some concerns	Low	Some concerns	Some concerns	Some concerns
Kużdzał et al. (35)	Low	Some concerns	Low	Some concerns	Some concerns	Some concerns
Santos et al. (23)	Some concerns	Some concerns	Low	Low	Some concerns	Some concerns

Table 4 presents the ROBINS-I assessment for the only non-randomized study (25). Overall risk of bias was judged critical, driven primarily by critical risk of confounding inherent to the uncontrolled pre–post design and additional concerns in participant selection, deviations from intended interventions, outcome measurement, and selective reporting.

**Table 4:** Risk of bias assessment in non-randomized trials.

Study	Confounding	Selection of participants	Classification of interventions	Deviations from intended interventions	Missing data	Measurement of outcomes	Selection of reported result	Overall ROBINS-I
Pavlović et al. (25)	Critical	Serious	Low	Serious	No information	Serious	Serious	Critical

### 3.4 Results of the included studies

Table 5 reports effects measured immediately after the intervention and within 0–12 h, capturing acute responses (e.g., pain, pressure pain threshold, mechanical muscle properties, range of motion, and hemodynamic variables).

**Table 5:** Effects of dry needling in short window (immediate and up to 12 h after intervention).

Study	Effects immediate after intervention	Effects 0–12 h	Main conclusions
Kużdzał et al. (35)	After 1st session (5 min): Muscle tone *p* = 0.007 (*d* = 1.00), CG > MTDN; Muscle stiffness *p* = 0.001 (*d* = 1.37), CG > MTDN; PPT *p* = 0.149, MTDN = CG; Fmax *p* = 0.550, MTDN = CG; Lateral-flexion ROM *p* = 0.348, MTDN = CG; Rotation ROM *p* = 0.504, MTDN = CG. After 3rd session (5 min): Muscle tone *p* < 0.001 (*d* = 1.50), CG > MTDN; Muscle stiffness *p* < 0.001 (*d* = 1.75), CG > MTDN; PPT *p* = 0.537, MTDN = CG; Fmax *p* = 0.706, MTDN = CG; Lateral-flexion ROM *p* = 0.975, MTDN = CG; Rotation ROM *p* = 0.784, MTDN = CG.	N.A.	Immediate: control better for tone/stiffness after sessions; most other outcomes (PPT, strength, ROM) showed no between-group differences.
Ceballos-Laita et al. (24)	Pain during throwing (NPRS): *p* < 0.001 (ES = 1.3), DN better than control; Internal rotation ROM: *p* < 0.001 (ES = 3.0), DN better; External rotation ROM: *p* = 0.025 (ES = 1.0), DN better; GIRD: *p* < 0.001 (ES = 2.6), DN better; ERG: *p* = 0.025 (ES = 0.3), DN better; Extensibility: *p* < 0.001 (ES = 2.9), DN better; Isometric strength (IR/ER): *p* > 0.05, DN = control.	N.A.	Immediate: DN improved pain during throwing and shoulder ROM measures (IR/ER, GIRD, ERG, extensibility) vs. no treatment; strength unchanged. These findings reflect an acute post-intervention context with maximal expectancy/measurement effects and do not establish next-session performance, functional recovery, or durability beyond the immediate window.
Aidar et al. (22)	Immediately post-recovery (0 min): SBP *p* < 0.001, DN < PR and DN < CW; DBP *p* > 0.05, DN = PR = CW; MBP *p* > 0.05, DN = PR = CW; HR *p* < 0.001, DN < PR (also CW < PR); DP *p* < 0.001, DN < PR (also CW < PR); MVO2 *p* < 0.001, DN < PR (also CW < PR).	50 min post-recovery: SBP *p* < 0.001, DN > PR and DN > CW; 60 min post-recovery: SBP *p* = 0.025, DN > PR and DN > CW. Other hemodynamic variables (DBP, MBP) *p* > 0.05 between methods across 0–60 min.	Immediate–0–12 h: DN reduced SBP immediately vs. PR/CW, but SBP was higher with DN at 50–60 min; HR/DP/MVO2 recovery favored DN/CW vs. PR.
Santos et al. (23)	15 min post-exercise (after recovery): MIF: time effect *p* = 0.001; method effect *p* = 0.046; interaction *p* = 0.002 (η2p = 0.25–0.39); post-hoc: MIF decreased vs. pretest after all recovery methods (pairwise DN vs. PR/CWI *p*-values not reported). PPT: increased immediately after all recovery methods (15 min); method effect *p* ≤ 0.003; interaction *p* ≤ 0.005 (η2p ≈ 0.39–0.51); pairwise DN vs. PR/CWI *p*-values not reported.	2 h post-exercise: MIF remained lower vs. pretest (reported decrease at 15 min and 2 h); PPT: after CWI, PPT increased significantly again at 2 h for pectoralis sternal part (pairwise p-values not reported).	Immediate–0–12 h: omnibus effects suggest recovery-method differences for MIF and PPT post-exercise; pairwise DN vs. PR/CWI *p*-values not consistently reported.
Ceballos-Laita et al. (32)	Stiffness *p* > 0.05, DN = DF (between-group Δchange −2.95 N/m; *d* = 0.1); Tone *p* > 0.05, DN = DF (Δchange 0.20 Hz; *d* = 0.4); Pain (VAS) *p* > 0.05, DN = DF (Δchange 0.45; *d* = 0.2); IR ROM p > 0.05, DN = DF (Δchange −1.18°; *d* = 0.1); ER ROM *p* > 0.05, DN = DF (Δchange 0.66°; *d* = 0.1); tROM *p* > 0.05, DN = DF (Δchange −0.52°; *d* = 0.1); HA ROM *p* > 0.05, DN = DF (Δchange −0.96°; *d* = 0.1); Extensibility *p* > 0.05, DN = DF (Δchange 1.22°; *d* = 0.1).	N.A.	Immediate: DN and DF showed no between-group differences for muscle properties, pain, ROM, or extensibility.
Pavlović et al. (25)	Pre vs. immediately after last treatment (session 4): SF-36 Physical functioning *p* = 0.011 (improved); Role-physical *p* = 0.001 (improved); Role-emotional *p* = 0.004 (improved); Social functioning *p* = 0.001 (improved); Pain *p* = 0.001 (improved); Mental health and Vitality *p* = 0.001 (improved); General health perception *p* ≈ 0.340 on average (no clear improvement). Self-rated health distribution improved *p* = 0.001.	N.A.	Immediate post-treatment (pre–post, no control): improvements reported in multiple SF-36 domains including pain and functioning.

CG: Control group; CW: Cold-water immersion; CWI: Cold-water immersion; DASH: Disabilities of the Arm, Shoulder and Hand questionnaire; DBP: Diastolic blood pressure; DF: Diacutaneous fibrolysis; DN: Dry needling; DP: Double product (HR × SBP); ERG: External rotation gain; ES: Effect size (as reported in study); Fmax: Maximal muscle contraction force (as reported in study); GIRD: Glenohumeral internal rotation deficit; HA: Horizontal adduction; HR: Heart rate; ICC: Intraclass correlation coefficient; IL: Interleukin; IL-2: Interleukin 2; IR: Internal rotation; ISP: Infraspinatus; LSST: Lateral scapular slide test; MBP: Mean blood pressure; MIF: Maximal isometric force; MTDN: Manual therapy + dry needling (as labeled in study); MTrP/MTrps: Myofascial trigger point(s); MVO2: Myocardial oxygen consumption estimate (as reported in study); NPRS: Numeric Pain Rating Score; NRS: Numeric Rating Scale; PPT: Pressure pain threshold; PR: Passive recovery; ROM: Range of motion; SBP: Systolic blood pressure; SF-36: 36-item Short Form Health Survey; tROM: Total rotation range of motion; UT: Upper trapezius; VAS: Visual Analogue Scale; N.A.: not applicable.

Table 6 reports effects from 12 h onward (12–24 h, 24–48 h, and >48 h), reflecting short-term follow-up responses and delayed effects where available.

**Table 6:** Effects of dry needling in late window (post 12 to >48 h after intervention).

Study	Effects 12–24 h	Effects 24–48 h	Effects >48 h	Main conclusions
Kużdzał et al. (35)	N.A.	N.A.	72 h after 3rd session: Muscle tone *p* = 0.002 (*d* = 1.23), CG > MTDN; Muscle stiffness *p* < 0.001 (*d* = 2.45), CG > MTDN; PPT *p* < 0.001 (*d* = 1.80), MTDN > CG; Fmax *p* = 0.791, MTDN = CG; Lateral-flexion ROM *p* = 0.142, MTDN = CG; Rotation ROM *p* = 0.896, MTDN = CG.	>48 h: delayed benefit for PPT at 72 h favoring MTDN; tone/stiffness still favored control; strength/ROM remained similar.
Kamali et al. (28)	N.A.	N.A.	Assessed 3 days after last session (3 sessions, 2-day interval): VAS *p* = 0.771, UT DN = ISP DN; DASH *p* = 0.520, UT DN = ISP DN; PPT *p* = 0.531, UT DN = ISP DN.	>48 h: UT DN and infraspinatus (remote) DN showed no between-group differences for pain (VAS), DASH, or PPT at 3 days post-treatment.
Santos et al. (23)	24 h post-exercise: MIF: PR and DN increased to baseline levels from 2 to 24 h (pairwise *p*-values not reported); PPT: lowest value noted 24 h after PR; DN showed similar pattern but generally smaller decrease than PR (pairwise *p*-values not reported).	48 h post-exercise: IL-2: recovery-method effect *p* < 0.006 (η2p = 0.47) and time effect *p* < 0.003 (η2p = 0.43); post-hoc: DN and CWI increased IL-2 from 24 to 48 h more than 2–24 h; PR showed no significant change over time (pairwise p-values not reported).	N.A.	12–48 h: IL-2 differed across recovery methods and time (omnibus effects), with DN/CWI showing higher IL-2 from 24–48 h than 2–24 h. However, the directionality of IL-2 as a better recovery signal is unclear and pairwise DN vs. comparator contrasts were often not reported. Force and PPT patterns were reported but were not consistently supported.
Ceballos-Laita et al. (32)	N.A.	N.A.	1 week follow-up: Stiffness *p* > 0.05, DN = DF (between-group Δchange −14.97 N/m; *d* = 0.5); Tone *p* > 0.05, DN = DF (Δchange −0.13 Hz; *d* = 0.2); Pain (VAS) *p* > 0.05, DN = DF (Δchange 0.53; *d* = 0.2); IR ROM p > 0.05, DN = DF (Δchange 0.63°; *d* = 0.1); ER ROM *p* > 0.05, DN = DF (Δchange 1.88°; *d* = 0.1); tROM *p* > 0.05, DN = DF (Δchange 2.50°; *d* = 0.1); HA ROM *p* > 0.05, DN = DF (Δchange 1.26°; *d* = 0.1); Extensibility *p* > 0.05, DN = DF (Δchange 2.41°; *d* = 0.2).	>48 h (1 week): DN and DF remained similar across outcomes; no between-group differences.

CG: Control group; CW: Cold-water immersion; CWI: Cold-water immersion; DASH: Disabilities of the Arm, Shoulder and Hand questionnaire; DF: Diacutaneous fibrolysis; DN: Dry needling; Fmax: Maximal muscle contraction force (as reported in study); HA: Horizontal adduction; IL: Interleukin; IL-2: Interleukin 2; IR: Internal rotation; ISP: Infraspinatus; LSST: Lateral scapular slide test; MIF: Maximal isometric force; MTDN: Manual therapy + dry needling (as labeled in study); MTrP/MTrps: Myofascial trigger point(s); NRS: Numeric Rating Scale; PPT: Pressure pain threshold; PR: Passive recovery; ROM: Range of motion; tROM: Total rotation range of motion; UT: Upper trapezius; VAS: Visual Analogue Scale.

## 4 Discussion

Across eight included studies, DN was investigated in sport and athlete populations primarily as a short-term intervention targeting recovery/readiness after strenuous exercise and pain- and function-related outcomes in athletes with regional myofascial pain or movement-related symptoms. Overall, the evidence suggests that DN may produce small-to-moderate short-term improvements in pain sensitivity and self-reported pain in some symptomatic athlete samples, with variable and often modest effects on range of motion (ROM) and performance-relevant outcomes. In recovery-focused crossover studies, DN was associated with changes in physiological recovery markers and selected tissue or biomarker outcomes, but the clinical and performance relevance of these changes remains uncertain due to heterogeneity in endpoints, short follow-up windows, and the absence of consistent readiness/performance confirmation. From a practice-translation perspective, performance relevance would ideally be demonstrated by changes that exceed measurement noise and align with athlete-facing endpoints, rather than isolated shifts in physiological proxies. For several proxies used in the included recovery trials (e.g., acute cytokine fluctuations and ultrasound-derived muscle thickness), direction-of-change is not inherently good or bad because post-exercise inflammatory and muscle-damage responses can reflect normal training adaptation rather than impaired recovery, thus proxy changes should be interpreted as signals unless corroborated by readiness/performance outcomes (26).

### 4.1 Recovery/readiness and physiological endpoints

Two randomized crossover investigations in Paralympic powerlifting evaluated DN as a post-exertion recovery strategy and compared DN against passive recovery and cold-water–based approaches, measuring hemodynamic indices and recovery-related physiological markers over short time horizons (22, 23). These studies contribute novel data in a highly trained population and benefit from within-subject crossover designs, which can increase efficiency when the condition is stable and carryover is controlled. However, the outcomes emphasize physiological proxies (e.g., hemodynamics, inflammatory markers) rather than validated readiness or return-to-performance endpoints, and sampling tends to cluster around acute windows (minutes to 48 h). As a result, even where DN differs from comparators on physiological trajectories, translation to sport-relevant outcomes (e.g., next-session performance, perceived readiness, competition availability) remains inferential rather than directly demonstrated (22, 23). To improve comparability and clinical interpretability, future DN recovery trials in athletes should adopt a core outcome set that includes (at minimum) a validated athlete-reported readiness/fatigue measure, DOMS/pain intensity (NRS/NPRS) at standardized timepoints, a next-session sport-relevant performance test (pre-specified and reliability-described), training/competition availability (missed sessions, modified training, or time-loss), and adverse events (standardized definitions and active surveillance) (27). Physiological proxies (e.g., cytokines, ultrasound thickness, hemodynamics) should be optional secondary outcomes, explicitly linked to hypotheses and interpreted alongside the core outcomes.

### 4.2 Pain intensity and pain sensitivity

A consistent theme across the symptomatic athlete trials is that DN can influence pain outcomes, including patient-reported pain and pressure pain threshold (PPT). In overhead-athlete and shoulder-region presentations, DN targeting relevant myofascial sites was associated with improvements in pain measures over short follow-up in some comparisons (24, 28, 29). The handball study targeting the teres major in elite athletes showed changes in pain during throwing alongside changes in shoulder-related measures immediately after intervention (24), supporting the plausibility of DN affecting pain-related responses in a sport-specific provocative task. Similarly, in overhead athletes with shoulder pain and scapular dyskinesis, DN combined with a comparator framework produced improvements in pain and PPT (29). Importantly, many included trials use active comparators rather than inert control, which is clinically relevant but tends to reduce apparent between-group effects and makes interpretation dependent on comparator efficacy and standardization. Accordingly, findings should be interpreted as absolute effects when DN is compared with sham/no intervention or incremental/added-value effects when DN is compared with an active standard. In our review, the strongest between-group signals were observed in the absolute (no-intervention) contrast, whereas incremental effects versus active comparators were generally smaller and more heterogeneous. Moreover, pain is a context-sensitive outcome influenced by expectation, co-interventions, and natural history, therefore, even when DN improves pain outcomes, the degree to which effects exceed placebo/context and whether they persist beyond early follow-up is not yet well established in athlete samples (28, 29).

### 4.3 Disability, function, and quality-of-life outcomes

Function/disability measures were reported in shoulder/overhead athlete contexts and generally moved in a favorable direction following DN interventions, though the magnitude and durability of change varied and was not always easily interpretable against minimal clinically important difference thresholds due to limited reporting of responder analyses (28, 29). Where anchors exist, practice translation would be strengthened by reporting the proportion of athletes achieving minimal clinically important difference MCID, not only mean differences, particularly in small samples where group means can mask clinically meaningful individual responses. For example, MCID estimates for numeric pain ratings in musculoskeletal pain are commonly on the order of 1–2 points, and MCID thresholds for DASH/QuickDASH are often in the 8–15-point range depending on population and anchor method, thus mapping observed changes to these thresholds would improve interpretability (30, 31).

A separate study assessed broader quality-of-life constructs in athletes with myofascial pain syndrome using questionnaire item responses pre/post DN and reported improvements across several items (25). While these findings are suggestive, single-group designs are particularly vulnerable to regression to the mean, expectancy effects, and time-related confounding. As such, these data should be interpreted as hypothesis-generating rather than confirmatory of DN’s effect on quality of life in athletes (25). Consistent with this, we did not use this uncontrolled pre–post study to infer a direction-of-effect for DN.

### 4.4 ROM and shoulder-specific mobility constructs

Multiple studies in overhead or throwing athletes assessed ROM and shoulder mobility constructs such as internal rotation (IR), external rotation (ER), total ROM, and GIRD-related measures. In the handball players treated at the teres major, DN was associated with changes in ROM and shoulder extensibility measures immediately post-intervention (24). In a later handball trial comparing DN with diacutaneous fibrolysis in the teres major region, both interventions showed changes across tissue mechanical properties and clinical outcomes, with between-group differences dependent on the specific endpoint and follow-up (32). These findings align with a broader clinical rationale that DN may modulate pain-related movement limitation and tissue sensitivity, which can secondarily influence ROM. A testable pathway is that DN reduces local pain sensitivity and increases stretch tolerance (perceptual/neuromotor restraint) rather than inducing immediate structural capsuloligamentous change (33). This would predict short-term ROM benefits that co-occur with reduced pain during provocative movement and normalize within days unless reinforced by loading/rehabilitation (33). However, ROM in overhead athletes is multifactorial (capsuloligamentous stiffness, neuromuscular control, training adaptation), and short-term ROM changes may not translate to improved performance or reduced injury risk without corroboration in longitudinal outcomes. In overhead sports, some ROM profiles (e.g., shifts in internal and external rotation, total arc) can represent adaptive trade-offs that support performance, and normalizing ROM toward non-thrower norms is not automatically desirable (34). Accordingly, ROM changes after DN should be interpreted relative to sport demands, symptoms, and task goals (e.g., pain-limited ROM vs. performance-adaptive ROM), and should not be presented as evidence of structural correction or injury prevention without prospective surveillance (24, 32). Accordingly, any injury prevention inference would require prospective injury surveillance (time-loss/recurrence) and exposure-adjusted designs, which were largely absent in the included literature.

### 4.5 Neuromuscular performance, strength, and tissue mechanical properties

The handball DN study reported changes in strength measures and posterior shoulder extensibility, suggesting that DN may influence neuromuscular output in the immediate window, potentially through pain modulation or altered muscle activation strategies (24). In the teres major comparison trial, myotonometry-derived stiffness and tone were included, expanding the evidence into quantifiable tissue property measures (32). In recovery-focused crossover work, markers such as maximal isometric force and muscle thickness were incorporated as part of the recovery profile (23).

### 4.6 Body region–specific effects

When findings are stratified by body region, the evidence base is concentrated in the shoulder/periscapular–cervical complex and the upper-limb musculature relevant to overhead sport, with fewer data addressing other anatomical regions. In overhead athletes and throwing-sport cohorts, DN targeting shoulder-related myofascial sites (e.g., teres major and periscapular trigger points) was most consistently associated with short-term improvements in pain-related outcomes and, less consistently, changes in shoulder mobility constructs (e.g., IR/ER ROM, extensibility) (24, 29, 32). Evidence for the neck and surrounding region was limited but suggests potential short-term benefit since in overhead athletes with shoulder symptoms, DN delivered to upper trapezius–related myofascial trigger points was associated with improvements in pain intensity and pressure pain threshold, alongside favorable changes in disability indices over short follow-up (28). By contrast, the recovery-focused crossover studies in Paralympic powerlifting used DN within an acute post-exercise context targeting muscles engaged in pressing/upper-limb force production, and reported changes in physiological recovery trajectories and selected tissue/biomarker endpoints, but these studies provide limited direct evidence that region-specific needling translates into sport performance or readiness at subsequent sessions (22, 23). Importantly, these samples included paraplegic athletes, and spinal cord injury is associated with autonomic and vascular alterations that can change blood pressure regulation, heart-rate recovery, and hemodynamic trajectories, therefore, generalizing hemodynamic recovery effects from Paralympic powerlifting to able-bodied athletes should be treated as an external-validity constraint (22). Thus the most reproducible signals appear in the shoulder-region symptom domain (pain/PPT), whereas evidence for meaningful region-specific effects on ROM normalization, strength restoration, or performance transfer remains inconsistent and should be interpreted cautiously.

### 4.7 Studies limitations

Across studies, the most recurrent source of some concerns in randomization process was insufficient reporting around allocation methods (e.g., limited detail on sequence generation and/or allocation concealment), even when baseline balance was acceptable. This pattern was observed in multiple trials in our sample (22, 23, 28, 29, 32). In parallel, measurement of the outcome frequently raised concerns because blinding of outcome assessors was unclear or not explicitly stated, and several outcomes relied on measures that can be influenced by expectations or assessor interaction. This contributed to some concerns in several studies and escalated to high risk in one trial where risk of differential or influenced outcome assessment was most plausible (24, 28, 29, 35).

Concerns were also concentrated in deviations from intended interventions and selection of the reported result. A typical issue was the practical impossibility of blinding therapists/providers in DN and manual therapy contexts, with limited information on whether deviations occurred and whether analyses aligned with the effect of assignment. This creates a plausible pathway for performance bias (differential co-intervention, encouragement, or dose modification) and co-intervention bias (additional care elements delivered unequally across arms), especially when outcomes are patient-reported or assessor-interactive. Mitigation strategies for future trials can be standardized co-intervention protocols with adherence reporting, introduce sham procedures with credibility assessment, blinded outcome assessors for all feasible endpoints, pre-specified primary outcomes/timepoints with publicly accessible analysis plans and prioritization of objective sport-relevant endpoints (e.g., attendance/availability, time-loss) alongside patient-reported measures.

This was particularly relevant for trials using hands-on interventions or sham procedures, where provider awareness and potential co-interventions could influence adherence or care pathways (22, 23, 32, 35). For selective reporting, some concerns commonly reflected the presence of multiple outcomes/timepoints and analytic choices combined with incomplete reporting of a finalized analysis plan, increasing the possibility of selective result selection even when trial registration existed (22–24, 28, 29, 35).

Consistent with this pattern of methodological limitations, the single non-randomized study was judged at critical overall risk of bias, largely because an uncontrolled pre–post design cannot adequately address confounding from natural recovery, regression to the mean, and expectancy/placebo effects (25). Consequently, its findings should be interpreted as hypothesis-generating and should not be pooled quantitatively with the randomized evidence without appropriate sensitivity or design-stratified analyses (25).

### 4.8 Future research and practical applications

Future trials should prioritize standardized DN protocols with transparent reporting of needle size, depth, targeting rationale, and dose, comparator frameworks that allow separation of specific and contextual effects (e.g., sham and active comparator arms where feasible), and longer follow-up where injury risk, training continuity, or sustained functional change are the target. For recovery studies, coupling physiological markers with validated readiness measures and next-session performance would substantially strengthen interpretability (22, 23). Future designs should explicitly distinguish outcomes (e.g., pain sensitivity/pressure pain threshold, neuromuscular control, localized tissue responses) from applied sport benefits (e.g., next-session performance, session completion/availability, time-loss outcomes), and power analyses should be aligned to the applied endpoint rather than to biomarker shifts alone.

Practically, DN may be considered as a time-limited adjunct for symptomatic athletes where short-term pain modulation and tolerable changes in ROM or function are desired, provided it is integrated into a broader rehabilitation and load-management plan rather than used as a stand-alone solution. Because mild adverse events (including post-treatment pain/soreness) are relatively common, DN should be trialed in lower-stakes training windows before competition, and avoided when prior responses suggest soreness could meaningfully degrade important sessions within the next 24–72 h (36). Teams should monitor individual response using a simple repeated approach (e.g., 0–10 soreness, symptom provocation on sport-specific tasks, and next-day session tolerance), and treat worsening symptoms or reduced task quality as a signal to modify dose, target, or timing rather than escalating exposure.

## 5 Conclusion

Overall, DN may be a reasonable adjunct for immediate-to-short-term (minutes to 1 week) symptom modulation in upper-quarter symptomatic athlete presentations represented in this review (overhead/throwing and handball samples), where outcomes primarily reflect pain/pain sensitivity and short-term ROM-related measures. By contrast, evidence that DN improves post-exertion recovery/readiness, next-session performance, or injury-risk endpoints (time-loss/recurrence/availability) remains sparse and indirect, and should not be inferred from isolated physiological proxy changes. Importantly, the current evidence does not justify solid claims that DN prevents injuries or produces consistent performance enhancement, because prospective injury surveillance and robust next-session performance/availability outcomes are largely absent and existing recovery trials rely on short-window proxies with uncertain directionality. Stronger, better-standardized trials are needed to clarify sport-relevant benefits and safety in real-world training and competition contexts.

## Data Availability

Data is available upon request to corresponding author.
